# Supplementary material for: Genome-wide identification, characterization and gene expression of BES1 transcription factor family in grapevine (Vitis vinifera L.)
Source: Sci Rep. 2023 Jan 5;13:240. doi: 10.1038/s41598-022-24407-y (PMC9816167; doi:10.1038/s41598-022-24407-y)
Supplement: Supplementary file 3 — Supplementary Information. [file 41598_2022_24407_MOESM3_ESM.zip › Vvi_Atr/Vitis_vinifera.PN40024.v4.dna_sm.toplevel.fa.vs.Amborella_trichopoda.AMTR1.0.dna_sm.toplevel.fa.html/Atr-AmTr_v1.0_scaffold00170.html]

|  |  |  |  |  |  |  |  |  |  |  |  |  |  |
| --- | --- | --- | --- | --- | --- | --- | --- | --- | --- | --- | --- | --- | --- |
| Duplication depth | Reference chromosome | Collinear blocks | | | | | | | | | | | |
| 0 | Atr-ERM98322 |  |  |  |  |  |  |  |  |  |  |  |  |
| 0 | Atr-ERM98323 |  |  |  |  |  |  |  |  |  |  |  |  |
| 0 | Atr-ERM98324 |  |  |  |  |  |  |  |  |  |  |  |  |
| 0 | Atr-ERM98325 |  |  |  |  |  |  |  |  |  |  |  |  |
| 1 | Atr-ERM98326 |  | Vvi-Vitvi19g00093\_t001 |  |  |  |  |  |  |  |  |  |  |
| 1 | Atr-ERM98327 |  | \| \| |  |  |  |  |  |  |  |  |  |  |
| 1 | Atr-ERM98328 |  | \| \| |  |  |  |  |  |  |  |  |  |  |
| 1 | Atr-ERM98329 |  | \| \| |  |  |  |  |  |  |  |  |  |  |
| 2 | Atr-ERM98330 |  | \| \| |  | Vvi-Vitvi10g00604\_t001 |  |  |  |  |  |  |  |  |
| 2 | Atr-ERM98331 |  | \| \| |  | Vvi-Vitvi10g00605\_t001 |  |  |  |  |  |  |  |  |
| 2 | Atr-ERM98332 |  | \| \| |  | \| \| |  |  |  |  |  |  |  |  |
| 2 | Atr-ERM98333 |  | \| \| |  | \| \| |  |  |  |  |  |  |  |  |
| 2 | Atr-ERM98334 |  | \| \| |  | \| \| |  |  |  |  |  |  |  |  |
| 2 | Atr-ERM98335 |  | \| \| |  | \| \| |  |  |  |  |  |  |  |  |
| 2 | Atr-ERM98336 |  | \| \| |  | \| \| |  |  |  |  |  |  |  |  |
| 2 | Atr-ERM98337 |  | \| \| |  | \| \| |  |  |  |  |  |  |  |  |
| 2 | Atr-ERM98338 |  | \| \| |  | \| \| |  |  |  |  |  |  |  |  |
| 2 | Atr-ERM98339 |  | \| \| |  | \| \| |  |  |  |  |  |  |  |  |
| 2 | Atr-ERM98340 |  | \| \| |  | Vvi-Vitvi10g00624\_t001 |  |  |  |  |  |  |  |  |
| 2 | Atr-ERM98341 |  | \| \| |  | \| \| |  |  |  |  |  |  |  |  |
| 2 | Atr-ERM98342 |  | \| \| |  | \| \| |  |  |  |  |  |  |  |  |
| 2 | Atr-ERM98343 |  | \| \| |  | \| \| |  |  |  |  |  |  |  |  |
| 2 | Atr-ERM98344 |  | Vvi-Vitvi19g00073\_t001 |  | \| \| |  |  |  |  |  |  |  |  |
| 2 | Atr-ERM98345 |  | \| \| |  | \| \| |  |  |  |  |  |  |  |  |
| 2 | Atr-ERM98346 |  | \| \| |  | Vvi-Vitvi10g00635\_t002 |  |  |  |  |  |  |  |  |
| 2 | Atr-ERM98347 |  | \| \| |  | \| \| |  |  |  |  |  |  |  |  |
| 2 | Atr-ERM98348 |  | Vvi-Vitvi19g00071\_t001 |  | \| \| |  |  |  |  |  |  |  |  |
| 2 | Atr-ERM98349 |  | \| \| |  | \| \| |  |  |  |  |  |  |  |  |
| 2 | Atr-ERM98350 |  | Vvi-Vitvi19g00061\_t001 |  | Vvi-Vitvi10g00636\_t001 |  |  |  |  |  |  |  |  |
| 2 | Atr-ERM98351 |  | Vvi-Vitvi19g00060\_t001 |  | Vvi-Vitvi10g00637\_t002 |  |  |  |  |  |  |  |  |
| 1 | Atr-ERM98352 |  | \| \| |  |  |  |  |  |  |  |  |  |  |
| 1 | Atr-ERM98353 |  | Vvi-Vitvi19g00059\_t001 |  |  |  |  |  |  |  |  |  |  |
| 0 | Atr-ERM98354 |  |  |  |  |  |  |  |  |  |  |  |  |
